# Potential Confounding of Diagnosis of Rabies in Patients with Recent Receipt of Intravenous Immune Globulin

**DOI:** 10.15585/mmwr.mm6705a3

**Published:** 2018-02-09

**Authors:** Neil M. Vora, Lillian A. Orciari, J Bradford Bertumen, Inger Damon, James A. Ellison, Vance G. Fowler, Richard Franka, Brett W. Petersen, P.S. Satheshkumar, Stephen M. Schexnayder, Todd G. Smith, Ryan M. Wallace, Susan Weinstein, Carl Williams, Pamela Yager, Michael Niezgoda

**Affiliations:** ^1^Division of High-Consequence Pathogens and Pathology, CDC; ^2^Epidemic Intelligence Service, CDC; ^3^Duke University, Durham, North Carolina; ^4^University of Arkansas for Medical Sciences, Little Rock, Arkansas; ^5^Arkansas Department of Health; ^6^North Carolina Department of Health and Human Services.

Rabies is an acute encephalitis that is nearly always fatal. It is caused by infection with viruses of the genus *Lyssavirus*, the most common of which is *Rabies lyssavirus*. The Council of State and Territorial Epidemiologists (CSTE) defines a confirmed human rabies case as an illness compatible with rabies that meets at least one of five different laboratory criteria.* Four of these criteria do not depend on the patient’s rabies vaccination status; however, the remaining criterion, “identification of *Lyssavirus*-specific antibody (i.e. by indirect fluorescent antibody…test or complete [*Rabies lyssavirus*] neutralization at 1:5 dilution) in the serum,” is only considered diagnostic in unvaccinated patients. *Lyssavirus*-specific antibodies include *Rabies lyssavirus*–specific binding immunoglobulin G (IgG) and immunoglobulin M (IgM) antibodies and *Rabies lyssavirus* neutralizing antibodies (RLNAs). This report describes six patients who were tested for rabies by CDC and who met CSTE criteria for confirmed human rabies because they had illnesses compatible with rabies, had not been vaccinated for rabies, and were found to have serum RLNAs (with complete *Rabies lyssavirus* neutralization at a serum dilution of 1:5). An additional four patients are described who were tested for rabies by CDC who were found to have serum RLNAs (with incomplete *Rabies lyssavirus* neutralization at a serum dilution of 1:5) despite having not been vaccinated for rabies. None of these 10 patients received a rabies diagnosis; rather, they were considered to have been passively immunized against rabies through recent receipt of intravenous immune globulin (IVIG). Serum RLNA test results should be interpreted with caution in patients who have not been vaccinated against rabies but who have recently received IVIG.

Rabies is preventable after a *Rabies lyssavirus* exposure through use of rabies postexposure prophylaxis; a standard rabies postexposure prophylaxis regimen in an immunocompetent patient who has not previously received rabies vaccination includes human rabies immune globulin and 4 doses of rabies vaccine (*1*). Human rabies immune globulin is prepared from plasma from human donors who have been hyperimmunized with rabies vaccine. It is delivered into wounds or intramuscularly to provide passive immunity during the time needed to develop an active immune response to vaccine antigen (*1*).^†^ IVIG is a blood product prepared from plasma of thousands of human donors who do not necessarily have a history of rabies vaccination (*2*). IVIG is administered to patients for a number of indications, including immunodeficiency states, neurologic disorders, infections, and autoimmune disorders. IVIG is not a component of rabies postexposure prophylaxis (*1*,*2*).

Data presented in this report were generated through routine clinical care and through testing of donated IVIG^§^ obtained from hospitals or manufacturers. Laboratory testing of patient specimens and IVIG was conducted at CDC and has been previously described (*3*). Nuchal skin biopsy was tested using the direct fluorescent antibody test for *Rabies lyssavirus* antigen. RNA was extracted and amplified from nuchal skin biopsy and saliva by reverse transcription–polymerase chain reaction targeting the *Rabies lyssavirus* nucleoprotein gene (*3*). Serum, cerebrospinal fluid, and IVIG were tested for *Rabies lyssavirus*–specific binding IgG and IgM antibodies using the indirect fluorescent antibody test and for RLNAs using the rapid fluorescent focus inhibition test (RFFIT).

## Case One

**FIGURE 1:**
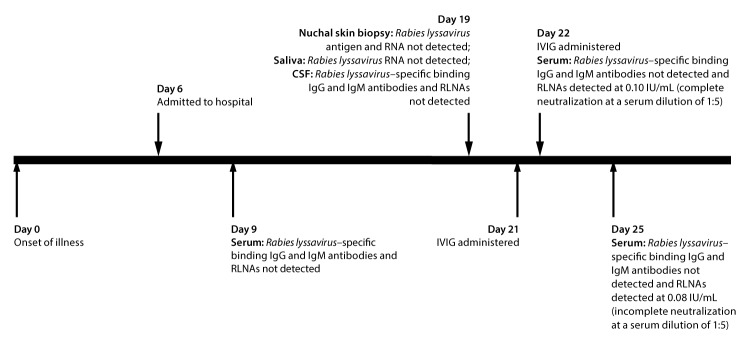
Timeline* of events for a patient with autoimmune encephalitis who met Council of State and Territorial Epidemiologists criteria for diagnosis of human rabies and had recently received intravenous immune globulin **Abbreviations:** CSF = cerebrospinal fluid; IgG = immunoglobulin G; IgM = immunoglobulin M; IVIG = intravenous immune globulin; RLNA = *Rabies lyssavirus* neutralizing antibody. * By number of days after illness onset.

In 2013, a previously healthy man in North Carolina, aged 28 years, with no prior history of rabies vaccination and no known recent mammal exposures experienced fever, body aches, headache, and neck stiffness (Figure 1). On the sixth day after illness onset (day 6), the patient was admitted to a hospital; shortly thereafter, he experienced seizures. He was initially treated empirically with antibiotics; IVIG (1 g/kg; Gamunex-C [Grifols, Los Angeles, California]) was administered on days 21 and 22. Rabies diagnostic testing was performed because of suspicion for rabies. Diagnostic testing did not reveal any evidence of *Rabies lyssavirus *infection, with the exception of detection of RLNAs by RFFIT (Table). RLNAs were not detected in serum collected on day 9, but were detected in sera collected on day 22 (0.10 international units [IU]/mL [complete *Rabies lyssavirus* neutralization at a serum dilution of 1:5]) and day 25 (0.08 IU/mL [incomplete *Rabies lyssavirus* neutralization at a serum dilution of 1:5]). Although *Rabies lyssavirus*–specific binding IgG antibodies were not detected in IVIG from the lots that the patient had received, RLNAs were detected at 0.45 IU/mL and 0.46 IU/mL (each lot demonstrated complete *Rabies lyssavirus* neutralization at an IVIG dilution of 1:5). Though the patient met CSTE criteria for confirmed human rabies (had an illness compatible with rabies, had not been vaccinated for rabies, and was found to have serum RLNAs [with complete *Rabies lyssavirus* neutralization at a serum dilution of 1:5]), his serum RLNAs were attributed to passive immunization against rabies through receipt of IVIG. Rabies was therefore ruled out and he received a diagnosis of autoimmune encephalitis. His illness improved after treatment.

**TABLE:** Characteristics and laboratory findings of unvaccinated patients in whom *Rabies lyssavirus* neutralizing antibodies were detected after receiving IVIG — nine states, 2013–2016

Case no.	Age (yrs)	Sex	Year testing was performed	State	Nuchal skin biopsy		Saliva	*Lyssavirus*-specific antibodies						Met CSTE rabies case definition^†^
								Cerebrospinal fluid			Serum (after receipt of IVIG)			
					*Rabies lyssavirus* antigen	*Rabies lyssavirus* RNA	*Rabies lyssavirus* RNA	IgG*	IgM*	RLNA (IU/mL)	IgG*	IgM*	RLNAs (IU/mL)	
1	28	M	2013	North Carolina	ND	ND	ND	ND	ND	ND	ND	ND	0.10^§^	Yes
2	13	M	2013	Arkansas	ND	ND	ND	ND	ND	ND	ND	ND	0.06^§^	No
3	13	M	2014	Texas	ND	ND	ND	ND	ND	ND	ND	ND	0.07	No
4	61	M	2015	South Carolina	ND	ND	ND	ND	ND	ND	ND	ND	0.08	No
5	38	M	2015	Maryland	IC^¶^	NT^¶^	NT^¶^	ND	ND	ND	ND	ND	0.07	No
6	11	M	2015	Texas	ND	ND	ND	NP	NP	NP	ND	ND	0.10	Yes
7	13	M	2015	Virginia	ND	ND	ND	ND	ND	ND	ND	ND	0.11	Yes
8	23	F	2015	Tennessee	ND	ND	ND	ND	ND	ND	ND	ND	0.18	Yes
9	40	M	2016	Massachusetts	NP	NP	ND	ND	ND	ND	ND	ND	0.20^§^	Yes
10	16	M	2016	Indiana	NT	ND	ND	ND	ND	ND	ND	ND	0.15	Yes

**Abbreviations:** CSTE = Council of State and Territorial Epidemiologists; F = female; IC = inconclusive; IgG = immunoglobulin G; IgM = immunoglobulin M; IVIG = intravenous immune globulin; M = male; ND = not detected; NP = not provided; NT = not tested; RLNA = *Rabies lyssavirus* neutralizing antibody.

* *Rabies lyssavirus*–specific binding immunoglobulin.

^†^ These patients met CSTE criteria for diagnosis of human rabies because they had illnesses compatible with rabies, had not been vaccinated for rabies, and had *Rabies lyssavirus* neutralizing antibodies with complete *Rabies lyssavirus* neutralization at a serum dilution of 1:5.

^§^ IVIG from the same lot(s) this patient had received was tested for *Rabies lyssavirus*–specific binding IgG antibodies and RLNAs; *Rabies lyssavirus*–specific binding IgG antibodies were not detected, but RLNAs were detected.

^¶^ Unsatisfactory sample.

## Case Two

**FIGURE 2:**
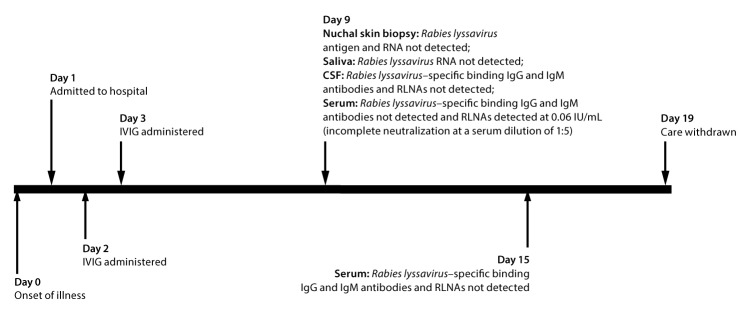
Timeline* of events for a patient with Eastern equine encephalitis virus infection who had no history of rabies vaccination, but in whom *Rabies lyssavirus* neutralizing antibodies were detected after receiving intravenous immune globulin **Abbreviations:** CSF = cerebrospinal fluid; IgG = immunoglobulin G; IgM = immunoglobulin M; IVIG = intravenous immune globulin; RLNA = *Rabies lyssavirus* neutralizing antibody. * By number of days after illness onset.

In 2013, a previously healthy male adolescent in Arkansas, aged 13 years, with no prior history of rabies vaccination and no known recent mammal exposures experienced 3 days of headache and three episodes of new-onset seizures (Figure 2). On the first day after illness onset (day 1), he was hospitalized. He was initially treated empirically with antibiotics; IVIG (1 g/kg; Gamunex-C [Grifols, Los Angeles, California]) was administered on days 2 and 3. Rabies diagnostic testing was performed because of suspicion for rabies. Diagnostic testing did not reveal any evidence of *Rabies lyssavirus *infection, with the exception of detection of RLNAs by RFFIT (Table). RLNAs were detected in serum collected on day 9 (0.06 IU/mL [incomplete *Rabies lyssavirus* neutralization at a serum dilution of 1:5]) but not on day 15. Although *Rabies lyssavirus*–specific binding IgG antibodies were not detected in IVIG from the lot that the patient had received, RLNAs were detected at 0.38 IU/mL (complete *Rabies lyssavirus* neutralization at an IVIG dilution of 1:5). Though the patient had serum RLNAs on day 9, *Rabies lyssavirus *neutralization at a serum dilution of 1:5 was incomplete, and he therefore did not meet CSTE criteria for confirmed human rabies. His serum RLNAs were attributed to passive immunization against rabies through receipt of IVIG. Rabies was therefore ruled out and additional diagnostic testing revealed Eastern equine encephalitis virus infection. Because of his poor prognosis, care was withdrawn and the patient subsequently died (*4*).

## Additional Cases

During 2014–2016, diagnostic testing of eight additional patients revealed a similar laboratory profile (Table). All eight patients had illnesses compatible with rabies, had no history of rabies vaccination, and had serum RLNAs (five with complete and three with incomplete *Rabies lyssavirus *neutralization at a serum dilution of 1:5). In each case, RLNAs were detected in sera collected only after IVIG administration. IVIG (Privigen [CSL Behring, King of Prussia, Pennsylvania]) was available from two lots received by one of these eight patients. RLNAs were detected in these two lots at 0.44 IU/mL and 2.3 IU/mL (each lot demonstrated complete *Rabies lyssavirus* neutralization at an IVIG dilution of 1:5). IVIG from lots that the remaining patients had received was unavailable for testing. None of these eight patients received a rabies diagnosis, including the five who met CSTE criteria for confirmed human rabies, because their serum RLNAs were attributed to passive immunization against rabies through receipt of IVIG.

## Results of Additional IVIG Testing

IVIG from a lot that had not been administered to any patient described here (Gammaplex [Bio Products Laboratory, Elstree, England]) was tested. Although *Rabies lyssavirus*–specific binding IgG antibodies were not detected, RLNAs were detected at 0.47 IU/mL (complete *Rabies lyssavirus* neutralization at an IVIG dilution of 1:5).

## Discussion

IVIG administration is known to confound serologic diagnosis of infections with pathogens such as human T-lymphotropic virus and *Toxoplasma* (*5*,*6*). This report describes 10 patients in which administration of IVIG confounded the diagnosis of human rabies. RLNAs were detected in serum from all 10 patients despite their never having been vaccinated for rabies, and it was ultimately determined that they had been passively immunized against rabies through receipt of IVIG. Six of these patients met CSTE criteria for human rabies because the concentration of serum RLNAs after IVIG administration was high enough to result in complete *Rabies lyssavirus* neutralization at a serum dilution of 1:5. However, in the absence of other laboratory evidence of rabies, and based on the knowledge that they had recently received IVIG, these patients all received alternative diagnoses.

Laboratory test results from the first patient are particularly illustrative. RLNAs can develop late in the course of illness in human rabies, but in this patient, it is likely that detection of serum RLNAs on days 22 and 25 (after IVIG administration), but not on day 9 (before IVIG administration), resulted from passive immunization through receipt of IVIG (administered on days 21 and 22) (*7*). Furthermore, serum RLNA concentration decreased as time passed after IVIG administration, as would be expected if these serum RLNAs were the result of passive immunity through IVIG, rather than part of an active immune response to a natural *Rabies lyssavirus* infection. A similar decline in serum RLNA concentration after IVIG administration was observed in the second patient.

These data suggest that detection of RLNAs in serum of an unvaccinated patient is a reliable laboratory criterion for human rabies only if IVIG has not been administered shortly before serum collection. If RLNAs are detected in serum collected after IVIG administration, additional testing of IVIG from the lot or lots used to treat the patient can be helpful in evaluating the likelihood of rabies.

There are several possible explanations for the detection of RLNAs in IVIG. First, because the IVIG that was tested was prepared by companies that also prepare human rabies immune globulin, the possibility exists that plasma from persons who were hyperimmunized with rabies vaccine also was used to prepare the IVIG that was tested (*8*).^¶^^,^** In addition, persons who donated plasma for IVIG preparation might have previously received rabies vaccination for a clinical indication (but were not hyperimmunized with rabies vaccine). IVIG from only six unique lots was tested and accurately evaluating variation in RLNA concentration from one IVIG preparation to the next was not possible, particularly because pathogen-specific antibodies are known to vary among IVIG preparations (*9*). Thus, although RLNAs might be present in IVIG, it is important that IVIG not be used as a replacement for human rabies immune globulin when administering rabies postexposure prophylaxis. Serum RLNA test results in patients who have not been vaccinated for rabies but who have recently received IVIG should be interpreted with caution when assessing whether a patient might have rabies.

**Summary**

**What is already known about this topic?**

The presence of a high concentration of serum *Rabies lyssavirus* neutralizing antibodies (RLNAs) in a patient with an illness compatible with rabies and no history of rabies vaccination is considered diagnostic for human rabies. This case definition does not take into account whether the patient has recently received intravenous immune globulin (IVIG).

**What is added by this report?**

This report describes six patients who met the case definition for human rabies because they had illnesses compatible with rabies, had not been vaccinated against rabies, and were found to have a high concentration of serum RLNAs. However, none of these patients received a rabies diagnosis; rather, they were considered to have been passively immunized for rabies through receipt of IVIG.

**What are the implications for public health practice?**

Positive RLNA test results should be interpreted with caution in a patient who has not been vaccinated against rabies but who has recently received IVIG. If RLNAs are detected in serum collected after IVIG administration, additional testing of IVIG from the lot or lots used to treat the patient can be helpful in evaluating the likelihood of rabies.

## Footnotes

* https://wwwn.cdc.gov/nndss/conditions/rabies-human/case-definition/2011/.

† http://www.grifolsusa.com/documents/10192/60862/ft_hyperrab_eeuu_EN/09f14ece-e450-48f8-9137-3ce7e0aaa8c6.

¶ https://www.medicines.org.uk:443/emc/PIL.14821.latest.pdf.

** https://primaryimmune.org/wp-content/uploads/2017/02/IVIG-Chart-2.2017.pdf.

## References

[1] Rupprecht CE, Briggs D, Brown CM, Use of a reduced (4-dose) vaccine schedule for postexposure prophylaxis to prevent human rabies: recommendations of the advisory committee on immunization practices. MMWR Recomm Rep 2010;59(No. RR-2). 20300058

[2] Jolles S, Sewell WA, Misbah SA. Clinical uses of intravenous immunoglobulin. Clin Exp Immunol 2005;142:1–11. 10.1111/j.1365-2249.2005.02834.x 16178850 PMC1809480

[3] Vora NM, Basavaraju SV, Feldman KA, Raccoon rabies virus variant transmission through solid organ transplantation. JAMA 2013;310:398–407. 10.1001/jama.2013.7986 23917290 PMC7552820

[4] Garlick J, Lee TJ, Shepherd P, Locally acquired Eastern equine encephalitis virus disease, Arkansas, USA. Emerg Infect Dis 2016;22:2216–7. 10.3201/eid2212.160844 27662563 PMC5189158

[5] Pelloux H, Fricker-Hidalgo H, Brochier G, Goullier-Fleuret A, Ambroise-Thomas P. Intravenous immunoglobulin therapy: confounding effects on serological screening for toxoplasmosis during pregnancy. J Clin Microbiol 1999;37:3423–4. 10488226 10.1128/jcm.37.10.3423-3424.1999 PMC85593

[6] Bélanger SS, Fish D, Kim J, Cohen S. False-positive human T-lymphotropic virus serology after intravenous immunoglobulin transfusion. CMAJ 2012;184:1709–12. 10.1503/cmaj.120019 22927508 PMC3478355

[7] CDC. Human rabies—Alabama, Tennessee, and Texas, 1994. MMWR Morb Mortal Wkly Rep 1995;44:269–72. 7708035

[8] Manning SE, Rupprecht CE, Fishbein D, Human rabies prevention—United States, 2008: recommendations of the Advisory Committee on Immunization Practices. MMWR Recomm Rep 2008;57(No. RR-3). 18496505

[9] Lamari F, Anastassiou ED, Tsegenidis T, Dimitracopoulos G, Karamanos NK. An enzyme immunoassay to determine the levels of specific antibodies toward bacterial surface antigens in human immunoglobulin preparations and blood serum. J Pharm Biomed Anal 1999;20:913–20. 10.1016/S0731-7085(99)00087-4 10746960

